# Correlates of concurrent partnerships and patterns of condom use among men who have sex with men and transgender women in Peru

**DOI:** 10.1371/journal.pone.0222114

**Published:** 2019-09-16

**Authors:** Angela K. Ulrich, Jorge Sanchez, Javier R. Lama, Lisa E. Manhart, Steven M. Goodreau, Ann C. Duerr

**Affiliations:** 1 Vaccine and Infectious Disease Division, Fred Hutchinson Cancer Research Center, Seattle, WA, United States of America; 2 Department of Epidemiology, University of Washington, Seattle, WA, United States of America; 3 Asociación Civil Impacta Salud y Educación, Lima, Peru; 4 Centro de Investigaciones Tecnológicas, Biomédicas y Medioambientales, Callao, Peru; 5 Department of Anthropology, University of Washington, Seattle, WA, United States of America; University of South Florida, UNITED STATES

## Abstract

**Background:**

In Peru, there is an ongoing high-incidence HIV epidemic among men who have sex with men (MSM) and transgender women (TW). Sexual concurrency, or having sex with a partner in between two acts of sex with another partner, may be a key factor in onward HIV transmission. In this study, we quantify concurrency, evaluate factors associated with concurrency, and assess condom use with concurrent partners among MSM and TW in Peru.

**Methods:**

We conducted a secondary analysis of data from the 2011 Peruvian Biobehavioral Survey. Pearson’s Chi-squared test was used to identify individual-level characteristics associated with concurrency. We estimated the association between participant characteristics, concurrent partnerships, partnership type (stable vs. non-stable), and CLAI within the context of concurrent partnerships using multivariate and repeated-measure Poisson regression.

**Results:**

3-month cumulative prevalence of concurrency was higher among TW compared to MSM (30.7% vs 25.2%, p = 0.014). Among those with concurrent stable and non-stable partners, 45% used condoms with both partners (95% CI: 40%-50%) and 30% preferentially had CLAI with the stable partner only (95%CI: 26%-35%). Factors associated with CLAI within the context of concurrent partnerships varied between MSM and TW.

**Conclusions:**

Although concurrency is common among TW and MSM in Peru, patterns of concurrency and differential condom use may vary between TW and MSM. Future research may explore differential condom use with stable and non-stable partners to better understand behavioral factors that may alter vulnerability to HIV in TW compared to MSM.

## Introduction

Worldwide, men who have sex with men (MSM) and transgender women (TW) are disproportionately burdened by HIV [1]. Biologic risk factors associated with MSM and TW compared to non-MSM and non-TW—among these a higher lifetime numbers of partners and a higher per-act probability of transmission of HIV during anal versus vaginal sex (1.4% vs 0.08%)—likely drive this disparity[2, 3]. Sexual networks also play an important role in HIV transmission; high connectivity is hypothesized to drive HIV epidemics, particularly in heterosexual populations [4–7]. Sexual concurrency, or the practice of having sex with a partner in between two acts of sex with another partner, increases the risk of HIV transmission, even beyond the risk associated with having multiple serial partners (i.e. one after another in time, with no overlap in partnerships) [4, 8–10]. Under serial monogamy, HIV can only be transmitted to partnerships beginning later in time, whereas under concurrency, HIV acquired from a partnership that began later in time can be transmitted back to a partnership that began earlier in time, if the earlier relationship is still ongoing. In other words, serial monogamy provides a “protective sequence” of partners, compared to having multiple partners that overlap in time. Furthermore, once an individual with concurrent partners acquires infection from one partner, transmission to the another concurrent partner(s) can occur without the potential delay involved in ending the previous relationship and starting a new one [11].

A number of studies from high income countries have described concurrency patterns among MSM, but little research has described concurrency in low-to-middle income countries, in Latin America, or among TW specifically. In the US and Australia there is evidence that compared with non-MSM, MSM are 2–3 times more likely to report sexual concurrency, and MSM are more likely to be in open relationships with a primary partner or to have an agreement in place that one or both partners may have other sex partners [12, 13]. Limited quantitative and qualitative research suggest that MSM in main partnerships have adopted behavioral strategies such that condomless anal intercourse (CLAI), even in the context of concurrent partnerships, carries little risk of HIV transmission[14, 15]. One such strategy, termed “negotiated safety,” typically requires two conditions: first, both partners must test negative for HIV and disclose their status, and second, any sex outside the main partnership will be “relatively safe” (e.g. use of condoms or refrain from anal sex)[14]. Since this study was conducted, pre-exposure prophylaxis (PrEP) has also been shown to be a highly effective biomedical option for HIV prevention among MSM[16]. At the time this study was conducted, however, PrEP was not available in Peru.

In Peru, there is an ongoing high-incidence HIV epidemic largely among MSM and TW. Between 2010–2016, new HIV infections increased by 24% in Peru, and gay men and other MSM had an estimated HIV prevalence over 15%[17]. In the capital city of Lima, where the majority of HIV cases are reported, HIV prevalence has been estimated to be as high as 22% among MSM and 30–49% among TW [18–20]. Since current prevention efforts are failing to reduce HIV incidence among MSM and TW, empirical estimates of the levels of sexual concurrency, correlates of concurrency, and information about the interaction of concurrency with CLAI can inform more targeted HIV prevention activities. The aims of this paper are to provide insight in the nature of concurrency and CLAI patterns among Peruvian MSM and TW by quantifying the level of concurrency, describing individual-level characteristics associated with concurrent partnerships, and identifying factors associated with condomless anal intercourse (CLAI) within the context of concurrent partnerships.

## Materials and methods

### Study population

This paper presents a secondary analysis of data collected between June and October 2011 as part of the Peruvian Biobehavioral Survey, a nationwide survey of MSM and TW. A total of 5,137 Peruvian MSM (n = 4,440, 86.4%) and TW (n = 697, 13.6%) from five Peruvian cities (Lima/Callao, Iquitos, Ica, Piura, Pucallpa) were enrolled and completed the questionnaire. Participants were eligible for the study if they were at least 18 years of age, assigned male sex at birth, and had at least one male or TW sexual partner in the previous 12 months. This convenience sample was recruited through posters, flyers, informational meetings, and outreach by peer educators at venues frequented by MSM and TW (e.g. saunas, adult movie theaters, video arcades, nightclubs, bars, beauty parlors, and sporting arenas). To reflect the sexually active population, the present analysis is limited to the 2,405 MSM and 414 TW who reported at least one male or TW partner in the three months preceding interview, and who had complete data regarding the dates of first and most recent sex with each partner.

### HIV testing and questionnaire

All participants underwent HIV testing with Determine HIV-1/2 third generation rapid antibody test (Alere Inc., MA, USA) and confirmed with Western blot. Pre- and post-test HIV counseling, risk reduction counseling, condoms, and lubricant were provided. Participants with sexually transmitted infections (STI) were managed according to Peruvian STI treatment guidelines and those diagnosed with HIV received standard health care following Peruvian HIV and AIDS health care management guidelines. Participants answered a questionnaire in the form of a 45-minute computer-assisted self-administered interview (CASI), which assessed demographics, alcohol and drug use, and sexual behaviors. The latter included the total number of male or TW sex partners in the three months preceding the interview, and specific questions regarding the three most recent male or TW partners.

### Partnership-level data

Questions regarding the three most recent male or TW sexual partners included the following: type(s) of sex acts (anal, oral), sexual role during anal sex (insertive, receptive), condom use during anal intercourse (yes, no), dates of first and most recent sex, and whether the respondent expected to have sex again in the future (yes, no, or don’t know). Respondents also identified whether the partner was a stable partner (*pareja stable*, “a person you live with, see often, or with whom you feel a special connection”) or a non-stable partner (either a *caserito*, “a person with whom you’ve had or are having sex, but you don’t consider him/her your partner”, or *vacilón/punto/agarre*, “a person with whom you’ve had sex only once”).

### Concurrency measures

Concurrency was calculated using methods recommended by UNAIDS[21]. According to these methods, we asked participants about the date of first sex (day, month, and year) and date of most recent sex (day, month, and year) with their three most recent sexual partners. These dates were used to calculate the duration of each partnership, and assess whether there was an overlap between partners. We calculated the 3-month cumulative prevalence of at least one instance of concurrency (proportion of participants with any overlap in the 3-months preceding interview) and the corresponding 95% confidence intervals.

### Covariates

Covariates included self-reported sociodemographic characteristics: residence (Lima vs. elsewhere), age quartile, education (any vs. no post-secondary education), sexual orientation (homosexual, heterosexual, or bisexual), gender (male-identified or transgender), sexual role (insertive, receptive, or versatile), and sex worker status (yes vs. no); HIV-status and HIV test history; alcohol use disorder (AUD) identified using the Alcohol Use Disorders Identification Test (AUDIT) [22], with an AUDIT score ≥8 indicating an AUD; and sexual behavior information including the total number of male and TW sexual partners in the previous three months, which was log transformed to more closely approximate a normal distribution.

### Negotiated safety

We did not ask a direct question about negotiated safety with sexual partners. Participants were considered to have behavior consistent with negotiated safety if condoms were used with the non-stable partner during a period of concurrency with a stable partner.

### Statistical analysis

We described sociodemographic and behavioral characteristics associated with concurrency in the three months preceding interview and used Pearson’s Chi-squared test to evaluate factors associated with concurrency. Bivariate analyses using Poisson regression with a log link and corresponding 95% confidence intervals were used to estimate the prevalence ratio (PR) of each covariate associated with concurrent partnerships (any vs. none). Poisson regression with robust standard errors was used in lieu of logistic regression because the outcome, concurrency, was common (>10%). Stepwise backward multivariate Poisson regression was used to estimate the adjusted prevalence ratio of concurrency associated with each variable. Covariates were considered for the full multivariate analysis if they were associated with concurrency in bivariate analyses (p-value <0.10) and maintained in the multivariate model if p≤0.05. In an attempt to identify characteristics associated with concurrency above and beyond those associated with having multiple partners, the multivariate analysis was adjusted for total number of male or TW partners in the previous three months. The final model was constructed by selecting the covariates that minimized both the Akaike information criterion (AIC) and the Bayesian information criterion (BIC).

As we were interested in differential condom use by partner type (stable vs. non-stable) among those with concurrent partners, we calculated the proportion of respondents with each combination of concurrent partners (two stable partners, two non-stable partners, or one stable and one non-stable partner), and the proportion of those with a concurrent stable and non-stable partner who reported CLAI. Two sets of bivariate analyses were conducted with generalized estimating equations (GEE) using Poisson regression to estimate the prevalence ratio of participant characteristics associated with 1) CLAI with a stable partner among those reporting a stable partner, and 2) CLAI with a non-stable partner among those reporting a non-stable partner. Since up to three dyads were possible for each respondent, estimates were adjusted for this within-person correlation structure. Although we hypothesized that there would be confounding by sociodemographic variables of age, education, income, role, and sex worker status, adjustment did not change the point estimate of the significant predictors, and no adjustment was made in the final analyses.

### Missing data

Participants for whom relational duration could not be calculated based on the information provided by the participant in the questionnaire were excluded from this analysis. We conducted a sensitivity analysis in which we imputed relational dates based on the following assumptions: for partnerships in which the date of first sexual encounter with a partner was reported but there was no date of most recent sexual encounter, the date of most recent sex was imputed based on the type of partner (stable, casual, or anonymous) and whether or not the respondent reported that s/he thought sex would occur again in the future. The sexual partnership was considered ongoing for stable and casual partners for whom it was unknown whether sex would occur in the future; the date of most recent sex was imputed as the midpoint between date of first sex and the date of their interview for stable partners with whom sex would not occur again; for anonymous partners and for casual partners whose relationship status was not ongoing, sex was considered a one-time event. The results from the analyses with imputed relational data were similar to the results from the analysis using only complete data, and thus only cases with complete data are presented in this paper.

### Software

All statistical analysis was performed using Stata 14.1 software [23].

### Ethical approval

Ethical approval for this study was obtained from the Fred Hutchinson Cancer Research Center in Seattle, WA (6007–613) and the Asociación Civil Impacta Salud y Educación, Lima, Peru (00140–2011).

## Results

### Participant characteristics

Of the 5,137 MSM and TW included in the national survey who completed the questionnaire, 3,949 reported at least one male or TW partner in the previous three months, and of these 3,019 provided complete relational timing data that allowed us to calculate concurrency status (Table 1). The characteristics of the 930 participants who reported at least one male/TW partner, but were missing complete relational timing data are presented in supplemental Appendix 1. The median age of respondents in this sample was 26 (IQR: 21–34), 14% were TW, and 63% identified as homosexual. In the three months preceding interview, participants reported a median of three male or TW sex partners in the previous three months (IQR: 1–6), and one-third had at least one stable partner and three-quarters had at least one non-stable partner. Nearly 8% (n = 241) screened positive for HIV and over 60% reported at least one previous HIV test.

**Table 1:** Sociodemographic and behavioral characteristics of participants reporting at least one male partner in the three months preceding interview (N = 3,019). P-values compare the proportion between groups (concurrency vs. no concurrency) and were calculated with Chi-squared tests.

	Total		Concurrent Partners		No Concurrent Partners		p-value
	N	(%^a^)	N	(%^b^)	N	(%^b^)	
Total	3019	(100)	783	(25.9)	2236	(74.1)	—
HIV Status							
Positive	241	(8.0)	61	(25.3)	180	(74.7)	
Negative	2778	(92.0)	722	(26.0)	2056	(74.0)	0.818
Previous HIV test							
Yes	1864	(61.7)	569	(30.5)	1295	(69.5)	
No	1155	(38.3)	214	(18.5)	941	(81.5)	<0.001
Location							
Lima	1477	(48.9)	421	(28.5)	1056	(71.5)	
Outside Lima	1542	(51.1)	362	(23.5)	1180	(76.5)	0.002
Age (years)							
≤21	809	(26.8)	154	(19.0)	655	(81.0)	
22–25	632	(20.9)	146	(23.1)	486	(76.9)	
26–31	664	(22.0)	174	(26.2)	490	(73.8)	
≥32	914	(30.3)	309	(33.8)	605	(66.2)	<0.001
Any Post-Secondary Education							
Yes	1168	(61.3)	347	(29.7)	821	(70.3)	
No	1851	(38.7)	436	(23.6)	1415	(76.4)	0.004
Income							
< Minimum Wage	2033	(67.3)	472	(23.2)	1561	(76.8)	
≥ Minimum Wage	986	(32.7)	311	(31.5)	675	(68.5)	<0.001
Sexual Orientation							
Homosexual	1897	(62.8)	573	(30.2)	1324	(69.8)	
Heterosexual	268	(8.9)	36	(13.4)	232	(86.6)	
Bisexual	853	(28.3)	174	(20.4)	679	(79.6)	<0.001
Gender							
Transgender Women	414	(13.7)	127	(30.7)	287	(69.3)	
Cisgender MSM	2605	(86.3)	656	(25.2)	1949	(74.8)	0.018
Sexual Role^c^							
Insertive	1011	(33.5)	179	(17.7)	832	(82.3)	
Receptive	1102	(36.5)	346	(31.4)	756	(68.6)	
Versatile	905	(30.0)	258	(28.5)	647	(71.5)	<0.001
Sex work							
Yes	587	(19.4)	186	(31.7)	401	(68.3)	
No	2432	(80.6)	597	(24.6)	1835	(75.5)	<0.001
Any Alcohol Use Disorder (AUDIT≥8)							
Yes	1905	(63.1)	508	(26.7)	1397	(73.3)	
No	1114	(36.9)	275	(24.7)	839	(75.3)	0.231
Total Number of Male Sex Partners							
Median (IQR)	3	(1–6)	4	(2–10)	2	(1–5)	<0.001
No. of stable partners in last 3 months							
0	1916	(63.5)	431	(22.5)	1485	(77.5)	
1	731	(24.2)	238	(32.6)	493	(67.4)	
2+	372	(12.3)	114	(30.7)	258	(69.4)	<0.001

^a^Column percent

^b^Row percent.

^c^One participant included in this table did not report their sexual role. All percentages are calculated from non-missing values.

### Prevalence and correlates of cumulative concurrency

The 3-month cumulative prevalence of concurrency in our study population was 25.9% (N = 783; 95% CI: 24.4–27.5%). Transgender participants were significantly more likely to report a concurrent partner in the previous three months compared to cisgender participants (30.7% vs 25.2%, p = 0.014). The total number of male partners in the previous three months was positively associated with having concurrent partners for both cisgender and transgender participants (Table 2). Among cisgender participants, a higher number of stable partners was associated with increased prevalence of concurrency, and among transgender participants, reporting one stable partner was associated with increased prevalence of concurrency compared to those who reported no stable partners (aPR = 1.63, 95% CI: 1.12–2.39). Cisgender participants in older age groups had a significantly higher prevalence of 3-month cumulative concurrency compared to those in the youngest age group. Among cisgender participants, those with concurrent partners were significantly more likely to have tested for HIV in the past, and to have reported condomless anal intercourse (aPR = 1.29, 95% CI: 1.10–1.50).

**Table 2:** Prevalence ratio associated with concurrent partnerships for cisgender and transgender participants. Results from unadjusted and adjusted Poisson regression.

	Cisgender MSM				Transgender Women			
	Unadjusted PR	(95% CI^b^)	Adjusted^a^ PR	(95% CI^b^)	Unadjusted PR	(95% CI^b^)	Adjusted^a^ PR	(95% CI^b^)
Log number of male/TW partners	1.16	(1.12–1.20)	1.15	(1.11–1.19)	1.10	(1.04–1.17)	1.09	(1.03–1.17)
HIV-positive	0.92	(0.63–1.70)			1.03	(0.63–1.70)		
Previous HIV test	1.64	(1.39–1.94)	1.25	(1.05–1.49)	1.49	(0.91–2.46)		
Unaware of HIV-positive status^c^	0.49	(0.23–1.01)			0.70	(0.20–2.42)		
Lima (Ref. = Outside Lima)	1.29	(1.11–1.51)			0.85	(0.60–1.20)		
Age (Ref. = ≤21 years)								
22–25	1.31	(1.04–1.66)	1.20	(0.94–1.52)	0.89	(0.52–1.52)	0.78	(0.45–1.35)
26–31	1.52	(1.21–1.92)	1.43	(1.13–1.81)	0.89	(0.51–1.54)	0.81	(0.46–1.41)
≥32	1.83	(1.47–2.27)	1.64	(1.31–2.06)	1.68	(1.04–2.69)	1.56	(0.97–2.52)
Any Post-Secondary Education	1.30	(1.11–1.51)			1.21	(0.83–1.77)		
Income ≥ Minimum Wage	1.36	(1.17–1.57)			1.68	(1.18–2.39)	1.43	(0.99–2.96)
Sexual Orientation (Ref = Heterosexual)								
Homosexual	2.20	(1.56–3.09)			^d^			
Bisexual	1.49	(1.04–2.14)			^d^			
Sexual Role (Ref. = Insertive)								
Receptive	1.73	(1.43–2.10)	1.44	(1.18–1.76)	1.30	(0.77–2.20)		
Versatile	1.61	(1.32–1.95)	1.29	(1.06–1.58)	^d^			
Sex work	1.22	(0.99–1.51)			1.34	(0.92–1.96)		
Any Alcohol Use Disorder	1.03	(0.88–1.21)			1.36	(0.90–2.06)		
# of stable partners in last 3 months (Ref = 0)								
1	1.39	(1.17–1.66)	1.31	(1.10–1.57)	1.64	(1.12–2.38)	1.63	(1.12–2.39)
2+	1.43	(1.14–1.79)	1.48	(1.18–1.86)	1.02	(0.61–1.74)	1.14	(0.67–1.95)
Any CLAI	1.49	(1.28–1.73)	1.29	(1.10–1.50)	1.21	(0.85–1.72)		

^a^Adjusted for all other variables presented in the adjusted column

^b^CI = Confidence interval

^c^Among those with an HIV-positive test

^d^Unstable estimates due to small numbers are not reported here.

### Condom use and concurrent partners

To test for evidence of negotiated safety, we explored condom-use patterns among participants who reported at least one instance of concurrency between a stable and non-stable partner. Participants reporting any concurrency (N = 783) provided detail on 1270 total overlapping partnerships. Combined, MSM and TW had 31% (95% CI: 29–34%) overlapping stable and non-stable partners, and a considerable proportion of participants with a concurrent partnership between a stable and non-stable partner had CLAI with both partners (20%, 95% CI: 16%-24%). A majority of partnerships showed behavior consistent with negotiated safety; either using condoms with both partners (45%, 95% CI: 40%-50%) or preferentially having CLAI with the stable partner only (30%, 95%CI:26%-35%). These proportions did not differ significantly between cisgender and transgender participants.

No factors we explored were significant predictors of CLAI with a stable partner among cisgender MSM (Table 3). Among transgender participants, those who had concurrency consisting of a stable and non-stable partner were significantly more likely to report CLAI with their one stable partner compared to those who had two concurrent stable partners (PR = 3.12, 95% CI: 1.13–8.63). Among cisgender participants, predictors of CLAI with a non-stable partner included alcohol use disorder (PR = 1.36, 95% CI: 1.06–1.75), and living in Lima (PR = 1.71, 95% CI: 1.34–2.17). Among cisgender participants with a non-stable partner, those with a concurrent stable partner were significantly less likely to have CLAI with the non-stable partner than those with two concurrent non-stable partners (PR = 0.57, 95% CI: 0.45–0.71). No factors we explored were significant predictors of CLAI with a nonstable partner among transgender participants.

**Table 3:** Prevalence ratio (PR) associated with CLAI with stable and non-stable partners among those with at least one instance of concurrent partners.

	CLAI with stable partner^a^				CLAI with a non-stable partner^b^			
	Cisgender MSM		Transgender women		Cisgender MSM		Transgender women	
	PR	(95% CI)	PR	(95% CI)	PR	(95% CI)	PR	(95% CI)
Total participants (n)	309		77		552		115	
Total partnerships (n)	464		120		897		186	
HIV-positive	0.90	(0.56–1.43)	1.15	(0.46–2.87)	0.66	(0.38–1.17)	0.96	(0.46–2.00)
Previous HIV test	1.22	(0.93–1.60)	1.16	(0.54–2.46)	0.84	(0.67–1.06)	1.49	(0.50–4.48)
Lima (Ref = outside Lima)	1.19	(0.94–1.51)	0.64	(0.35–1.17)	1.71	(1.34–2.17)	1.73	(0.98–3.07)
Age (Ref ≤21 years)								
22–25	1.20	(0.81–1.78)	1.00	(0.44–2.31)	1.15	(0.83–1.59)	1.83	(0.37–8.99)
26–31	0.99	(0.63–1.55)	0.82	(0.32–2.10)	1.09	(0.78–1.51)	1.17	(0.20–6.74)
≥32	0.97	(0.65–1.46)	1.21	(0.58–2.55)	1.07	(0.79–1.46)	2.42	(0.53–11.1)
Any Post-Secondary Education	1.01	(0.80–1.27)	0.69	(0.30–1.59)	1.06	(0.86–1.31)	1.28	(0.71–2.32)
Income ≥Min Wage	1.26	(1.00–1.60)	0.78	(0.43–1.44)	1.00	(0.80–1.25)	0.59	(0.31–1.11)
Sexual Orientation (Ref = Heterosexual)								
Homosexual	1.21	(0.55–2.71)	^c^		0.80	(0.50–1.29)	^c^	
Bisexual	1.06	(0.46–2.46)	^c^		0.85	(0.51–1.40)	^c^	
Sexual Role (Ref = Insertive)								
Receptive	1.08	(0.76–1.53)	^c^		0.85	(0.63–1.14)	^c^	
Versatile	1.23	(0.88–1.73)	^c^		1.14	(0.87–1.49)	1.42	(0.77–2.63)
Sex work	1.00	(0.72–1.40)	1.20	(0.61–2.46)	1.22	(0.93–1.61)	0.97	(0.52–1.79)
Any Alcohol Use Disorder	1.25	(0.98–1.61)	1.24	(0.53–2.92)	1.36	(1.06–1.75)	1.19	(0.52–2.66)
Combination of concurrent partners (Ref = both stable partners)								
1 stable, 1 non-stable partner	1.03	(0.82–1.30)	3.12	(1.13–8.63)	N/A		N/A	
Combination of concurrent partners (Ref = both non-stable partners)								
1 stable, 1 non-stable partner	N/A		N/A		0.56	(0.44–0.72)	0.63	(0.37–1.09)

^a^Conditional upon having at least one stable partner

^b^Conditional upon having at least one non-stable partner.

^c^Unstable estimates due to small numbers not reported here.

## Discussion

This study used data from the 2011 Peruvian Biobehavioral Survey to quantify concurrency, to evaluate factors associated with concurrency, and to assess condom use with concurrent partners among MSM and TW in Peru. We found that while both Peruvian MSM and TW have a high prevalence of concurrent sexual partners, TW reported significantly higher cumulative 3-month prevalence of concurrency compared to cisgender MSM (30% vs 25%, respectively). Our estimate of concurrency among MSM is comparable to previous estimates of concurrency in other MSM populations in Western countries,[24, 25] and higher than estimates from heterosexual populations in sub-Saharan Africa[26]. Our study is one of few to specifically report the prevalence of concurrency in TW populations, and suggests that patterns of concurrency and condom use may be different among TW compared to MSM. Historically in the HIV literature, data regarding MSM and TW have been conflated; however, analyzing TW and MSM as separate populations improves the ability to identify distinct social or behavioral factors that may increase vulnerability to sexually transmitted infections, including HIV[27].

This study elucidated the convergence of transmission-related risk factors, particularly among MSM: alcohol use disorder (AUD), condomless anal intercourse (CLAI), and concurrent sexual partnerships. A substantial proportion (20%) with concurrent partners did not use condoms with either their stable or non-stable partner, consistent with previous studies which found a considerable proportion of the population is unlikely to use condoms with any partner, even if these partners overlap in time [25]. Among MSM, AUD was associated with CLAI among people with non-stable partners. This is aligned with literature that shows alcohol and drug use are associated with concurrency and other sexual risk behavior [28–30], and studies from Peruvian populations showing AUDs are associated with risky sexual behavior including CLAI, anal sex in venues (e.g. saunas, nightclubs), sex with casual partners, and diagnosis of STIs[31]. Alcohol reduction interventions targeted to those with AUDs may serve as an indirect HIV-prevention strategy and may be particularly well suited for this high-risk population[32].

Of importance, the participants in our study displayed behavior consistent with HIV prevention approaches, including consistent condom use and knowledge of HIV status. Nearly half of the respondents in this study used condoms consistently with both stable and non-stable partners, and individuals in concurrent partnerships were relatively more knowledgeable of their HIV status and were more likely to have been previously tested for HIV.

Additionally, we hypothesized that there would be evidence in this population of negotiated safety, a practice in which main partners mutually agree to use condoms with non-main partners in order to honor a commitment to their primary partner and to protect themselves from STIs, including HIV [15]. Although this survey did not ask participants directly about their motivations for condom use, our data suggest that condoms are used differentially with stable compared to non-stable partners in concurrent partnerships. Among those who used condoms differentially by partner type, the majority either had CLAI with only the stable partner or with neither partner; on the other hand, only a small proportion had CLAI with only their non-stable partner. Our data also suggest that condom use with concurrent partners may be different between MSM and TW. Among MSM, CLAI with a non-stable partner was less common among those with a concurrent stable partner compared to those whose concurrent partner was non-stable. Among TW, CLAI with a stable partner was more common among those with a concurrent non-stable partner compared to those with two concurrent stable partners. Future research should explicitly ask about motivations for condom use with stable and non-stable partners in order to better interpret these findings.

It is possible that negotiated safety could be an effective risk reduction technique if practiced consistently and at high levels in these populations, but further modeling studies and empirical data is needed to confirm this hypothesis. In the absence of these data, condom use and frequent HIV testing may be appropriate and acceptable risk reduction techniques for both MSM and TW populations, given the already observed high-level uptake of these behaviors. Given the high proportion of MSM and TW with concurrent partners, and the known impact of concurrency in driving HIV epidemics, consideration of pre-exposure prophylaxis (PrEP) for those with concurrent partners, particularly those who do not use condoms consistently, may also be appropriate.

The results of this study should be interpreted with the following limitations in mind. First, individuals were only included in this analysis if they had complete data on first and most recent dates of sex with up to their three most recent partners. It is possible that those with missing data are systematically different from those with complete data, resulting in a biased estimate of concurrency. However, a sensitivity analysis with imputed dates suggests that the inference drawn from this study is robust. Second, the population included in the Biobehavioral Surveillance was a convenience sample and may not be representative of the general MSM and TW population. Recruitment was conducted at social venues, including bars and nightclubs, and at venues frequented by sex workers, possibly resulting in higher levels of alcohol use and risky sexual behavior. On the other hand, individuals sampled for participation in this study had a lower than expected HIV prevalence (8%), compared to over 20% prevalence in previous studies.[18–20] This could be due to the fact that those at higher risk had tested positive for HIV previously, and therefore chose not to participate in a screening study. Finally, PrEP was not available at the time this study was conducted but has since considerably changed the landscape of HIV prevention [16]. Although PrEP is not yet widely available in Peru, or in Latin America, it will change the definition of what is considered “safe” in both monogamous and concurrent partnerships as it is rolled out as a prevention strategy.

In conclusion, our study suggests that concurrency is common, but that patterns of concurrency and condom use may be different between TW and MSM. Future research should explore differential condom use with stable and non-stable partners to better understand behavioral factors that may alter vulnerability to HIV in TW compared to MSM. In the meantime, targeted interventions, such as frequent HIV testing, treatment of alcohol use disorder, and PrEP for HIV-negative individuals with concurrent partners may be appropriate to reduce HIV acquisition and transmission in both MSM and TW.

## Supporting information

S1 Appendix Sociodemographic characteristics of participants with non-missing and missing relational timing data. (DOCX)
